# Differences in *MB-COMT* DNA methylation in monozygotic twins on phenotypic indicators of impulsivity

**DOI:** 10.3389/fgene.2022.1067276

**Published:** 2023-01-06

**Authors:** Snežana Smederevac, Lissette Delgado-Cruzata, Dušanka Mitrović, Bojana M. Dinić, Toni-Ann T. Bravo, Maria Delgado, Vojislava Bugarski Ignjatović, Selka Sadiković, Ilija Milovanović, Nataša Vučinić, Bojan Branovački, Mechthild Prinz, Zoran Budimlija, Jelena Kušić‐Tišma, Željka Nikolašević

**Affiliations:** ^1^ Department of Psychology, Faculty of Philosophy, University of Novi Sad, Novi Sad, Serbia; ^2^ John Jay College of Criminal Justice, New York, NY, United States; ^3^ Mount Holyoke College, South Hadley, MA, United States; ^4^ Faculty of Medicine, University of Novi Sad, Novi Sad, Serbia; ^5^ Department of Neurology, School of Medicine, New York University, New York City, NY, United States; ^6^ Institute of Molecular Genetics and Genetic Engineering, University of Belgrade, Belgrade, Serbia

**Keywords:** MB-COMT, DNA methylation, monozygotic twins, risk behavior, aggression, personality, executive functions (EFs), impulsivity

## Abstract

Epigenetic modifications of the membrane bound catechol-O-methyltransferase (*MB*-*COMT*) gene may affect the enzymatic degradation of dopamine, and consequently, human behavior. This study investigated the association between membrane bound catechol-O-methyltransferase DNA methylation (DNAm) differences in 92 monozygotic (MZ) twins with phenotypic manifestations of cognitive, behavioral, and personality indicators associated with reward-related behaviors and lack of control. We used pyrosequencing to determine DNAm of the regulatory region of membrane bound catechol-O-methyltransferase in saliva DNA. Results of intrapair differences in the percentage of membrane bound catechol-O-methyltransferase DNAm at each of five CpG sites show that there are associations between phenotypic indicators of lack of control and membrane bound catechol-O-methyltransferase DNAm differences on CpG1, CpG2 and CpG4, suggesting the common epigenetic patterns for personality traits, cognitive functions, and risk behaviors.

## Introduction

An important contribution to the understanding of individual differences in behavior is provided by studies of epigenetic processes, such as the methylation of DNA sequences, which actively regulate the expression of the genetic information present in the human genome (Roth, 2013). In certain genetic regions, cytosines adjacent to guanines in CpG dinucleotides can be methylated and in this way limit access of the transcription machinery to the gene sequence. A growing body of evidence suggests that environmental stimuli can modulate DNA methylation (DNAm) patterns and the corresponding levels of expression in various genes, establishing a connection between environmental factors and biological effects including behavior (Abdolmaleky et al., 2004). From a genetic and neurophysiological perspective, many psychological traits, such as impulsivity and related behavior, are linked to changes in the dopaminergic system (DeYoung, 2013). However, an important challenge for the identification of potential susceptibility genes for impulsive behaviors arises due to different results between candidate gene studies (CGAS) and genome-wide association studies (GWAS). For example, many CGAS have found associations between aggression and dopaminergic genes, such as *DAT*, *DRD2*, *DRD4*, *COMT*, while none of *GWAS* related to these phenomena achieved genome-wide significance (Fernàndez-Castillo & Cormand, 2016). Moreover, CGA studies have shown importance of the interaction between *COMT* and *DRD2* genes, suggesting that high *COMT* activity and low D_2_ receptor density is associated with high impulsivity scores in smokers (Reuter et al., 2006), while such findings have not yet been confirmed in GWAS. In general, given the conservative GWAS significance threshold, they are underpowered to detect most associated SNPs. For example, the GWAS study of personality traits showed only six replicable genetic variants associated with personality, and five of which are novel (Lo et al., 2016). Although previous CGAS studies have suggested potential associations between personality traits, cognitive functions, risk behaviors, and dopamine genes (Reuter et al., 2006; Soeiro-De-Souza et al., 2013; Gurvich & Rossell, 2015; Fernàndez-Castillo & Cormand, 2016), an important research question addressed in this study relates to the association between different indicators of impulsivity and changes in DNAm in genes encoding proteins with important function in behavior.

### *MB-COMT* gene and dopamine regulation

Genes responsible for dopamine metabolism, transport and cellular reception have an important function in regulating all reward-related behaviors (DeYoung, 2013) include the catechol-O-methyltransferase (*MB-COMT*) gene, located on chromosome 22q11-q12, that plays a substantial role in the enzymatic degradation of dopamine (Dickinson & Elvevåg, 2009). *MB-COMT* is found in high concentrations within the prefrontal cortex of the brain and it has been found to determine personality, behavior inhibition, thinking, short-term memory, and emotion regulation (Chen et al., 2004; Abdolmaleky et al., 2006). *MB-COMT*-regulated processes account for approximately 60% of metabolic degradation of dopamine (Karoum et al., 1994), and phenotypic features commonly associated with *MB-COMT* enzyme activity include personality traits, cognitive abilities, and various behavioral indicators of lack of control (Dreher et al., 2008; Wacker et al., 2012; DeYoung, 2013). Most studies investigating variation in *MB-COMT* activity have looked into the presence of specific polymorphisms in this gene, rs4680, which encodes the substitution of valine (Val) by methionine (Met) at codon 158 (Val158Met substitution, see Rakvåg et al., 2008). The Met158 allele is strongly associated with lower abundance, stability, and activity of the *MB-COMT* enzyme. Carriers of the Val variant have a higher rate of dopamine degradation and this may be associated with the urge to constantly activate the reward system (Chen et al., 2004; Dreher et al., 2008). A recent meta-analysis on the functionality of dopamine gene variants has shown that *MB-COMT* Val158Met polymorphism has the clearest functional associations with dopamine regulation (Tunbridge et al., 2019). In addition to genetic variation, some studies have suggested that differential DNAm around the transcription start site of *MB-COMT* can play an important role on this gene’s expression and ultimately, its activity levels (Schreiner et al., 2011).

### *MB-COMT* gene and executive functions

Studying in the field of neuroepigenetics could provide valuable information regarding the specific mechanisms by which a gene is expressed or silenced in relation to neuropsychological phenotypes. Executive function refers to a set of interconnected processes required for a purposeful, goal-oriented behavior (Anderson, 2002; Hughes & Graham, 2002; Anderson et al., 2008; Miyake & Friedman, 2012; Diamond, 2013). Since this highly complex and integrated set of cognitive abilities includes many cognitive processes, such as planning, goal setting, task initiation, task monitoring, ability to inhibit or delay responses, evaluation of responses, cognitive flexibility, and selection of efficient strategies necessary for problem-solving (Luria 1966; Welsh et al., 1991; Zelazo et al., 1997; Anderson, 2002), their study relies on different methods, frequently without a clear conceptual or empirical framework. Previous research has shown an association between executive dysfunction and impulsivity (Bickel et al., 2012), suggesting that impulsivity may result from a failure or dysfunction of the executive system. Although some studies found an association between the *MB-COMT* promoter methylation and prefrontal activity (Ursini et al., 2011; Córdova-Palomera et al., 2014; Walton et al., 2014; Lewis et al., 2019), several others reported contradictory results (Gomes et al., 2012; Schiepers et al., 2012). More specifically, while studying healthy controls Ursini et al. (2011) found an association between DNA methylation at *S-COMT* and prefrontal activity during a working memory task. This study found that methylation of the high-activity Val allele is inversely related to *MB-COMT* expression and therefore partially compensates for its negative effects on prefrontal cognition and activity. Moreover, DNAm differences in *MB-COMT, DBH*, the dopamine transporter gene (*DAT1*) and two dopamine receptor genes (*DRD1* and *DRD2*) were associated with twin differences in inhibitory control (Lewis et al., 2019). However, in another study of older individuals with ages between 50–70 years, the global DNAm profile of leukocytes was not associated with cognitive function in the domains of memory, sensorimotor speed, complex speed, information speed and word fluency (Schiepers et al., 2012).

### *MB-COMT* gene and aggression

Previous research supported the hypothesis that *MB-COMT* genotypes modify the sensitivity of the environment that confers either risk or protective factors for aggression (Wang et al., 2018). The effect of gene-environment interaction is also highlighted in the recent meta-analysis that showed significant relationships between aggression and blood DNAm levels that were associated with expression levels of various genes previously linked to problematic behaviors (van Dongen et al., 2021). Moreover, the same DNAm levels associated with aggression were also associated with other risk behaviors (e.g., smoking, alcohol consumption and other chemical exposures, see van Dongen et al., 2021). In the comparison of blood DNAm levels within aggression-discordant MZ twin pairs, van Dongen et al. (2015) found no genome-wide significant DNAm differences, but they mapped three top ranking sites located near different genes. Importantly, these sites had a low heritability (up to 10%) and showed mean within-pair difference in DNAm percentage of 0.8%–1.8% (van Dongen et al., 2021). While this study did not identify *MB-COMT* as an important gene, previous genetic studies have identified associations of polymorphisms in this gene with various risk behaviors, including aggression (for a review see Fernàndez-Castillo & Cormand, 2016). This suggests it is important to further explore the role of *MB-COMT* DNAm on these behaviors.

### MB-COMT gene and personality traits

The revised reinforcement sensitivity theory (rRST; Gray and McNaughton, 2000), the most prominent conceptual frameworks in the field of “personality neuroscience”, provides plausible explanations of reactions to signals of reward and punishment, as well as reactions to the estimated danger in the form of fight, flight or freeze. Within the rRST, the Behavioral Inhibition System (BIS) is defined as the basis for the processing of conflicting stimuli, corresponding to anxiety; the Behavioral Approach System (BAS) is responsible for reactions to all appetitive stimuli, corresponding to impulsivity. Fight/Flight/Freeze system (FFFS) is the fear-related, underlying, defensive reaction to present threats, in the form of reactions such as confrontation, escape, or a temporary blocking (e.g., Gray and McNaughton, 2000). The dopamine system contributes to cognitive, sensory, and social perception, as well as cognitive and emotional processing of stimuli, which are key to responding to environments, according to rRST theory. Therefore, it is reasonable to assume that personality traits which are likely to be partially regulated by the *MB-COMT* gene, such as impulsivity (BAS) (Smederevac et al., 2022) and reactive aggression (Fight) as a response to a perceived threat (Reuter et al., 2006), will also be associated with differences in the level of *MB-COMT* DNAm. Moreover, previous studies have shown evidence that associate *MB-COMT* DNAm with personality disorders (Dammann et al., 2011; Thomas et al., 2019).

### Current study

Different research designs can be used to examine epigenetic changes, but the most significant advantage is derived from the co-twin design, which includes monozygotic twins (MZ twins). That design is based on the assumption that MZ twins share 100% of their DNA sequence (i.e., also DNA outside genes), and all the differences between them can be attributed to the influence of environmental factors. However, recent evidence points to the fact that the DNA of some MZ twins is not identical (e.g., Jonsson et al., 2021), which raises important methodological and conceptual questions for behavioral genetics. The effect of environmental factors, which can accumulate over time, resulting in significant phenotypic differences between MZ twins in adulthood (Fraga et al., 2005), as well as differences in their epigenome (Van Dongen et al., 2021). The main goal of the present study is to determine associations between differences in DNAm in the promoter region of the *MB-COMT* gene and differences in phenotypic manifestations associated with reward-related behaviors and lack of control between MZ twins. Previous studies have shown that impulsivity is a multidimensional construct, covering indicators such as impulsive choices, impulsive actions, and impulsive personality traits (MacKillop et al., 2016). Therefore, we used various psychological measures as cognitive and behavioral indicators of lack of control, as well as personality dimensions defined by the rRST (Gray and McNaughton, 2000) to encompass a wide range of phenotypic features. Personality traits defined by rRST represent a comprehensive measure of strategies for responding to environmental challenges, which may contribute to epigenetic changes. Indicators of cognitive aspects of control are WCST (Heaton et al., 1993) measures of executive functions, while behavioral indicators are represented by the frequency of risk behaviors, such as cigarette, alcohol, or drug abuse, as well as aggression under provocation induced by Competitive Reaction Time Task (CRTT; Warburton and Bushman, 2019). We hypothesized that differences between MZ twins on various indicators of lack of control would be associated with significant differences in *MB-COMT* gene DNAm. This assumption is possible for all phenotypic indicators that can be expressed as continuous measures, such as personality traits, executive functions, and aggressive reactions. However, since the study includes phenotypic characteristics that have different manifestations, we assumed that objectively measurable indicators, such as risky behaviors, can be examined by determining concordant and discordant MZ pairs, and by studying the association between intrapair phenotypic concordance/discordance and intrapair differences in DNAm on five *MB-COMT* gene sites.

## Materials and methods

### Sample

The entire procedure for recruitment, testing, and data collection within Serbian Twin Advanced Registry (STAR) is described elsewhere (Smederevac et al., 2019). From the STAR Registry which contains data on 1,654 participants (827 twin pairs), all monozygotic twin pairs with data on all relevant phenotypic measures and *MB-COMT* (rs4680) genotyping were selected. After excluding some cases due to failed genotyping, this sample consisted of 432 twins. To investigate whether DNAm differences were associated with behavioral phenotypic features, we selected a subsample of 92 (46 pairs) monozygotic twins (MZ) for whom buccal swab DNA was of good quality for methylation analysis. The population studied here includes 24 male and 68 female twins. The age of the participants ranged from 18 to 44 years old, with a mean age of 23.38 ± 6.28 years old. Due to incomplete data for some twins, the number of twin pairs depended on the analysis. Participation was voluntary and for all participants informed written consent have been obtained prior to the participating in the study. The research was approved by the Institutional Ethical Committees (codes: #02-374/15, #01-39/229/1, #O-EO-024/2020).

### Phenotypic measures

#### Reinforcement sensitivity questionnaire

Reinforcement Sensitivity Questionnaire (RSQ; Smederevac et al., 2014) is based on the Revised Reinforcement Sensitivity Theory (Gray & McNaughton, 2000) and contains 29 items distributed amongst five scales: Behavioral inhibition system—BIS (7 items, *α* = 0.65), Behavioral activation system—BAS (6 items, *α* = 0.75), and Fight/Flight/Freeze system—FFS (with 5 items each, *α* = 0.81, 0.44, 0.61, respectively). Items are rated on a four-point Likert scale (1 = *completely disagree* to 4 = *completely agree*).

#### Wisconsin card sorting test

Wisconsin Card Sorting Test (WCST; Heaton et al., 1993) is the most prominent test for the assessment of set-shifting, attention, and inhibition. The test assesses the possibility of creating and changing the principles of categorization, using the task of classifying a series of cards according to one of the three classification criteria: color, form, and a number of elements. The variables used in this study were: number of categories completed, number of perseverative errors, number of non-perseverative errors, and failures to maintain set.

#### Competitive reaction time task

Competitive Reaction Time Task (CRTT; Warburton and Bushman, 2019) is an experimental procedure for aggression induction. Throughout the procedure, twins were led to believe they were competing with each other in the reaction time tasks. Before each task, each twin had an opportunity to set the “punishment” for his/her twin pair. The punishment consisted of the settled intensity (on a scale from 0 = *no punishment*, 1 = 60 db to 10 = 105 db) and duration of an aversive noise (on a scale from 1 = 0.5 s to 10 = 5 s, see Dinić et al., 2020). After the punishment was established, the researchers began the competition. The slower twin received the punishment determined by the faster, winning twin. There were four blocks in the procedure (each contained 10 trials), with the first block designed as practice in which twins only administered the punishment and did not receive it if they were slower. In blocks 2 through 4, twins received predetermined punishments, which increased during the procedure: in the second block *M*_intensity_ = 70 db (60–75) and *M*_duration_ = 0.75 s (0.5–1); in the third block *M*_intensity_ = 85 db (80–90) and *M*_duration_ = 2 s (1.5–3); and in the fourth block *M*_intensity_ = 100 db (95–105) and *M*_duration_ = 4.2 s (3.5–5). In each block, twins randomly won in 50% of the tasks. Settled punishment in the 1st block refers to unprovoked aggression and the rest of the blocks to provoked aggression, punishment intensity refers to explicit, direct physical aggression and punishment duration to implicit, indirect aggression, although these outcomes could be combined (e.g., Giancola and Chermack, 1998).

#### Data on risk behaviors

Risk behaviors were assessed using three self-reported questionnaires about cigarette smoking, alcohol, and drug consumption. Participants also answered whether or not they consume alcohol, use drugs, and smoke cigarettes. The answers were categorized as 1 (yes) and 0 (no).

### Zygosity analysis

Zygosity was determined using Investigator 24plex GO! Kit (Qiagen®, Valencia, CA, USA). DNA swab analysis was tested by STR multiplex amplification of the CODIS and ESS loci, SE33, DYS391 and Amelogenin without prior DNA extraction. Kit detects 21 autosomal and two gender markers, Amelogenin and DYS391. Samples with partial profiles were interpreted if at least 10 loci had results. The amplified loci then underwent capillary electrophoresis in the Applied Biosystems 3,500 Genetic Analyzer. The results were analyzed using Applied Biosystems GeneMapper ID-X software.

### The genotyping of MB-COMT polymorphisms

The genotyping of the *MB-COMT* gene (rs4680) was carried out using TaqMan assays (TaqMan SNP, Applied Biosystems®, Warrington, United Kingdom), as recommended by the manufacturer. The TaqMan SNP Genotyping Assays uses TaqMan 5′ -nuclease chemistry for amplifying and detecting specific polymorphisms in purified genomic DNA samples and takes advantage of minor groove-binding probes for superior allelic discrimination.

Genotyping Assays contain a VIC-dye-labelled probe, a FAM-dye-labelled probe with two target-specific primers. PCR was performed using 10 ng of genomic DNA together with 1 µL of TaqMan Genotyping assay and 12.5 µL of the genotyping master mix in the final 25 µL reaction on a 96-well plate using an ABI Prism 7,500 Fast PCR device (Applied Biosystems®, Foster City, California, USA). *MB-COMT* gene (rs4680) alleles with the specific fluorescence curves were detected and analyzed using the 7,500 System SDS program, integrated into the ABI Prism 7,500 Fast PCR device.

The *MB-COMT* gene polymorphism was defined by 3 groups (Table 1): 130 high-activity homozygotes (Met/Met carriers), 225 intermediate heterozygotes (Met/Val carriers), and 77 low-activity homozygotes (Val/Val carriers). The MB-COMT gene polymorphism was in the Hardy-Weinberg equilibrium (*HWE*), with no significant differences between the observed and calculated genotype frequencies (χ^2^ = 4.13, df = 2, *p* > 0.05). However, results were the same for the *HWE* based on one member of a MZ twin pair [χ^2^ (df) = .913 (2); *p* > 0.05]. In actual sample the distribution of polymorphisms replicates the structure of the population from which it is sampled [χ^2^ (df) = .900 (2); *p* > .05].

**TABLE 1:** Genotype frequencies of *MB-COMT* Val158Met.

*MB/COMT*	Full sample	DNAm sample
Met/Met	130 (30,1%)	32 (34,8%)
Met/Val	225 (52,1%)	46 (50,0%)
Val/Val	77 (17,8%)	14 (15,2%)

### DNA methylation assay

**FIGURE 1:**
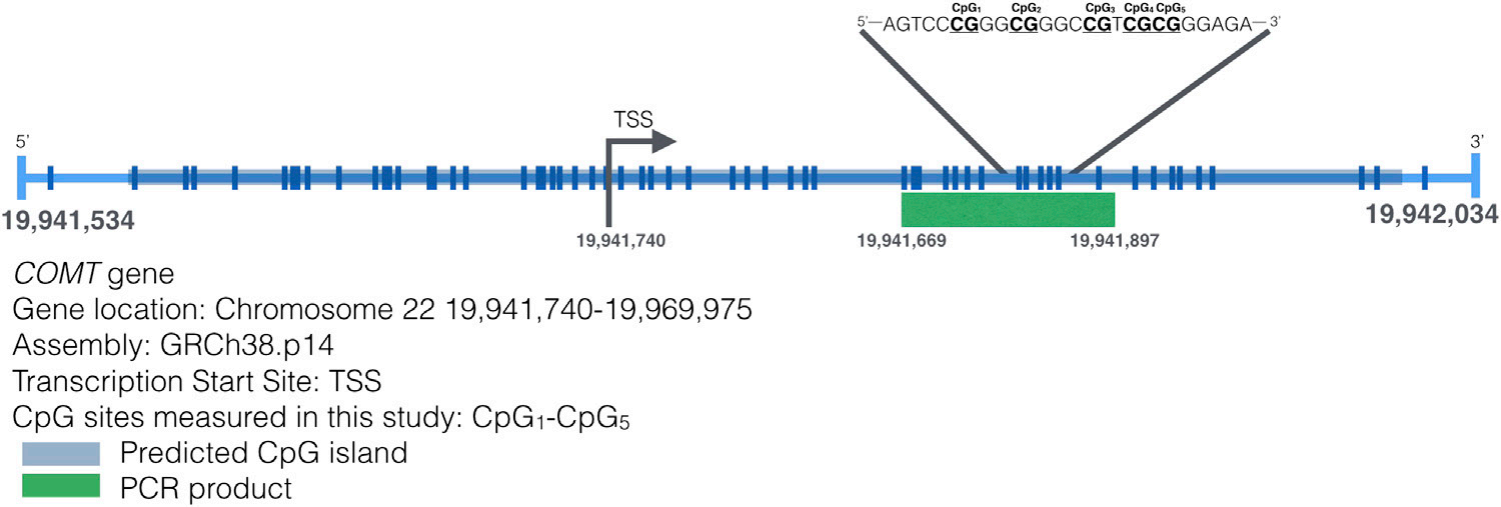
Map of the transcription start site of the *MB-COMT* gene and the CpG sites on that region. A 500bp sequence around the TSS of the *COMT* gene is presented. Lines represent CpG sites in the sequence. The inset highlights a sequence that includes the CpG sites measured in this study.

Genomic DNA was extracted out of buccal swabs using QIAamp DNA Mini Kit (Qiagen®, Valencia, CA, USA) according to the manufacturer’s extraction protocol. DNA was bisulfite converted using the EZ DNA Methylation-Gold kit (Zymo Research) following the kit’s protocol. Bisulfite-converted DNA was eluted in 15 μL of water and 2.5–5 μL were used for Pyromark PCR amplification (Qiagen). The assay targets a 228-bp DNA fragment in the *MB-COMT* promoter region (McGregor, 2014) (Figure 1). The forward primer had the following sequence 5′-TGG​GGT​AGA​TTA​GGG​TTG​T-3′ and the reverse primer biotinylated in the 5′ end was 5′-CCA​CAC​CCT​ATC​CCA​ATA​TTC-3′. Amplification conditions used were as recommended by the Pyromark PCR Kit (Qiagen). Pyrosequencing was carried out in a PyroMark Q24 (Qiagen) following the manufacturer’s instructions using 5′-GGA​TAG​GGG​AGG​GTT​TAG​TT-3′ as the sequencing primer and the following sequence to analyze 5′-TYGGGYGGGTYGTYGYGGGAGAGGTGAGAG-3′. Pyrosequencing measured DNA methylation levels of five CpG sites indicated in Figure 1. DNA methylation analysis was performed using the PyroMark Q24 Advanced 3.0.0 software. DNA methylation status was reported as the average percent methylation for the four CpG sites, or DNA methylation level at an individual CpG sites as indicated in each analysis. Each amplification and pyrosequencing run included fully methylated and unmethylated DNA (Zymo Research) as controls. No-template controls were also included in all runs.

### Statistical analysis

First, Friedman test was used to determine differences in DNA methylation percentage between individual CpG sites (data has marked deviations from normality).

Second, we conducted preliminary analyses to determine possible sex and age effects in phenotypic measures in order to apply appropriate correction of the scores. Phenotypes did not show a significant association with sex and age, except for three cognitive measures (percentage of responses at the conceptual level, perseverative errors, and non-perseverative errors) in which a significant sex effect was found. Thus, according to Bouchard and McGue (2003) regression technique, scores of these cognitive measures were partialized by sex and used in further analyses.

Next, given the sample size, we focused exclusively on the examination of differences between twins, avoiding analyzes that would lead to false positive findings, as they require much larger sample. Therefore, intrapair differences in the percentage of *MB-COMT* DNAm at each CpG site and mean CpG at all CpGs were calculated. For the analysis of associations with continuous phenotypic measures (personality and cognitive measures as well as laboratory-induced aggression), the differences in raw scores between twins in each pair were calculated. For the analysis of associations with categorical phenotypic measures (risk behaviors), we separated the twin pairs into two groups: twins that differ in the specific phenotype (discordant) versus twins that do not differ in the phenotype (concordant). For example, concordant twins are if both abuse or both do not abuse alcohol. Discordant are those in which one abuses and the other does not abuse alcohol.

Both skewness and kurtosis of these differences were positive and showed a violation of normal distribution (they are in a range from 3.88 to 6.60, i.e., outside the recommended range of ± 2 for a normal distribution, see Gravetter & Wallnau, 2017). Among intrapair differences in cognitive measures, all except non-perseverative errors showed high kurtosis (from 2.70 to 4.75), while intrapair differences in personality and aggression measures showed non-violation of normal distribution. Considering violation of normal distribution of intrapair differences in the *MB-COMT* DNAm and some phenotypic measures as well as that our sample is small, non-parametric tests in all analyses were applied. The associations between intrapair differences in continuous phenotypic measures and intrapair differences in DNAm levels were examined by computing Spearman rank correlation coefficients. Also, Mann-Whitney U-test was applied to determine differences between discordant and concordant twin pairs in intrapair differences in DNAm levels. To reduce Type I error, Bonferroni *p*-adjustment was calculated for applied 6 tests for each phenotypic measure (five CpG sites and mean CpG sites) as 0.05/6 = 0.0083. All analyses were conducted in SPSS IBM for Windows v.26 (IBM Corp, 2019).

## Results

### DNAm analysis and descriptive statistics

**FIGURE 2:**
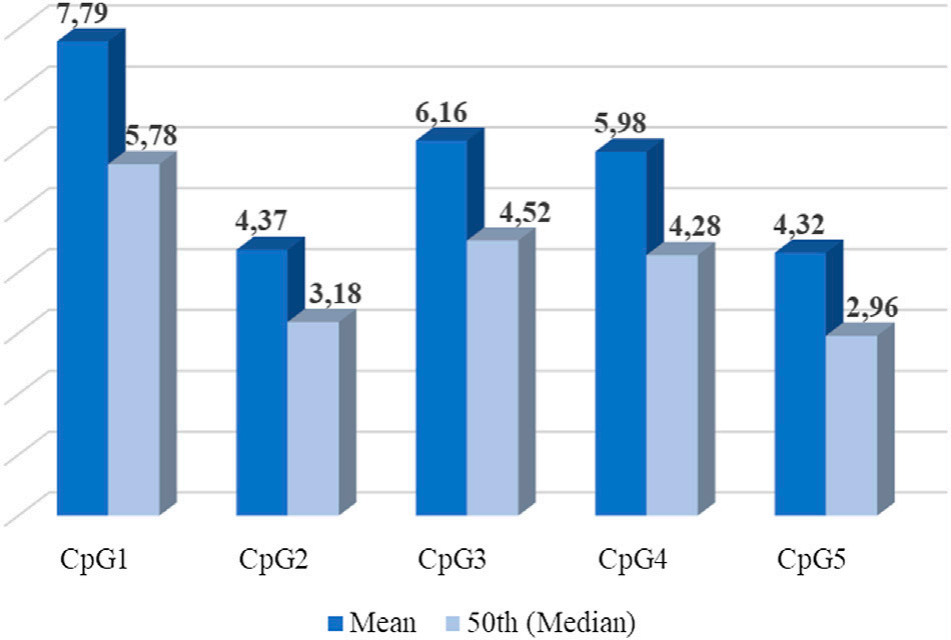
*MB-COMT* DNA methylation percentage among sites (Average percentage of DNA methylation for each of five CpG sites-mean and median).

Five CpG sites were identified in the selected fragment (Chr22:19941740-19969975) of the promoter methylation region of the *MB-COMT* gene, as shown in Figure 1. There were significant differences across CpG sites—c^2^ (4, *n* = 92) = 159,36, *p* < 0.001, and in Figure 2 the mean and median of the percentage of DNA methylation for each of the five CpG sites are shown.

CpG1 had a higher DNA methylation percentage than the other sites. Other pairwise comparisons are shown in Table 2.

**TABLE 2:** Differences between the DNAm levels of the five CpG sites using Durbin-Conover test.

	Statistic	*p*
CpG1-CpG2	12.50	<0.001
CpG1-CpG3	5.99	<0.001
CpG1-CpG4	4.11	<0.001
CpG1-CpG5	14.14	<0.001
CpG2-CpG3	6.51	<0.001
CpG2-CpG4	8.40	<0.001
CpG2-CpG5	1.64	0.103
CpG3-CpG4	1.88	0.061
CpG3-CpG5	8.15	<0.001
CpG4-CpG5	10.03	<0.001

Descriptive data of the percentage of *MB-COMT* DNAm and the phenotypic measures, intrapair differences in all measures, and the twin intrapair correlations are presented in Table 3. The intrapair correlations between MZ twins in all CpG sites are not significant, indicating no association in *MB-COMT* DNAm levels among twins (Table 3). The mean DNAm intrapair difference between MZ twins was around 4% (in a range between 3.88% and 4.94%) for all CpG sites, except for CpG 1, where the overall intrapair difference between MZ twins was 6.20 ± 6.60%. The intrapair correlations in cognition measures were not significant, indicating that MZ twins are not similar in specific aspects of cognitive functioning; however, interpair correlations in other phenotypic measures such as personality dimensions and laboratory-induced aggression were positive, significant, and of moderate to high strength.

**TABLE 3:** Descriptive statistics and twin correlation coefficients for *MB-COMT* DNAm and all phenotypic measures.

Measures		Twin 1	Twin 2	Differences	Intrapair correlations
		*M (SD)*	*M (SD)*		
*MB-COMT* DNAm (%)	CpG1	7.54 (5.56)	8.05 (7.42)	6.20 (6.60)	0.13
	CpG2	3.97 (3.70)	4.77 (5.20)	4.03 (5.39)	0.06
	CpG3	5.89 (4.31)	6.44 (6.26)	4.94 (5.75)	0.12
	CpG4	6.08 (4.63)	5.88 (5.56)	4.69 (5.77)	0.14
	CpG5	3.74 (3.25)	4.90 (5.05)	3.88 (4.88)	0.19
	Mean of all CpG sites	5.44 (3.98)	5.99 (5.59)	4.30 (5.43)	0.19
Cognition measures	CLR	69.76 (17.79)	74.71 (17.19)	15.19 (14.84)	0.20
	PE	13.28 (10.71)	9.34 (7.43)	7.41 (9.52)	0.15
	NPE	9.82 (7.01)	11.38 (10.35)	7.36 (7.51)	0.22
	FMS	0.79 (1.52)	0.56 (1.12)	0.79 (1.15)	0.12
Personality measures	BIS	15.69 (3.65)	14.77 (3.60)	3.69 (3.11)	0.28
	BAS	16.97 (3.97)	16.33 (3.43)	3.25 (2.61)	0.37*
	Fight	13.91 (4.06)	15.52 (3.81)	3.36 (2.71)	0.58**
	Flight	12.74 (2.59)	12.42 (2.64)	2.32 (2.02)	0.20
	Freeze	10.24 (2.99)	9.77 (2.43)	1.96 (2.03)	0.51**
Laboratory-induced aggression: punishment intensity	Block 1 = no provocation	4.78 (2.98)	4.71 (2.93)	0.70 (0.64)	0.54**
	Block 2 = low provocation	4.64 (3.08)	4.93 (3.25)	0.80 (0.59)	0.48**
	Block 3 = mild provocation	4.85 (3.18)	5.29 (3.34)	0.71 (0.51)	0.60**
	Block 4 = high provocation	5.13 (3.31)	5.64 (3.32)	0.77 (0.52)	0.55**
Laboratory-induced aggression: punishment duration	Block 1 = no provocation	4.28 (3.02)	4.49 (2.52)	0.83 (0.69)	0.41**
	Block 2 = low provocation	4.05 (2.78)	4.58 (2.87)	0.91 (0.66)	0.35*
	Block 3 = mild provocation	4.58 (3.13)	5.13 (3.04)	0.82 (0.61)	0.45**
	Block 4 = high provocation	4.86 (3.18)	5.68 (3.02)	0.75 (0.54)	0.56**

Note. CLR, Conceptual level response; PE, Perseverative errors; NPE, Non-perseverative errors; FMS, Failure to maintain set. **p < 0.01, **p < 0.05.

### Relationship between intrapair differences in MZ twins on continuous variables (cognitive measures, laboratory-induced aggression, and personality traits) and DNAm

Results of the correlations between intrapair differences in the percentage of *MB-COMT* DNAm and intrapair differences in cognitive measures showed that the only significant, positive and moderate correlation after the *p*-adjustment was obtained between differences in CpG site 1and differences in failures to maintain set (*p* = 0.005) (Table 4), pointing to the association between differences in CpG site 1 DNAm level and the differences in the phenotypic expression of this cognitive function. In the case of correlations with intrapair differences in aggression, two significant, positive and moderate correlations were found which remained significant after the Bonferroni *p*-adjustment. The significant correlations were found between intrapair differences in *MB-COMT* DNAm at CpG site 1 (*p* = 0.014) and CpG site 3 (*p* = 0.012) and intrapair differences in punishment intensity in block 1 of the CRTT procedure (direct unprovoked aggression). Thus, results indicated that differences in the levels of DNAm are associated with differences in the phenotypic expression of direct unprovoked aggression. Regarding correlations with personality dimensions, intrapair differences in the *MB-COMT* DNAm showed significant, positive and moderate to high correlations with intrapair differences in Freeze at CpG site 2 (*p* = 0.004), CpG site 3 (*p* = 0.019) and CpG site 4 (*p* = 0.007). However, only correlations at CpG site 2 and CpG site 4 remained significant after the *p*-adjustment. In addition, intrapair differences in *MB-COMT* DNAm at CpG site 4 showed a moderate positive correlation with intrapair differences in Fight, but this correlation was not significant after the *p*-adjustment (*p* = 0.041).

**TABLE 4:** Spearman’s rho coefficients between differences in CpGs and psychological measures.

Cognitive measures N=(39 MZ twin pairs)					Laboratory-induced aggression N=(42 MZ twin pairs)								Personality measures N = (28 MZ twin pairs)				
					Punishment intensity				Punishment duration								
DNAm difference (%)	Percent conceptual level responses	Perseverative errors	Non-perseverative errors	Failures to maintain set	Block 1 = no provocation	Block 2 = low provocation	Block 3 = mild provocation	Block 4 = high provocation	Block 1 = no provocation	Block 2 = low provocation	Block 3 = mild provocation	Block 4 = high provocation	BIS	BAS	Fight	Flight	Freeze
CpG1	0.08	−0.02	0.35*	0.44**	0.38*	0.17	0.10	0.12	0.06	−0.03	0.03	0.12	0.07	0.08	0.34	−0.16	0.27
CpG2	0.18	−0.15	0.13	0.22	0.12	−0.13	−0.01	−0.01	−0.01	−0.17	0.07	0.10	0.27	0.11	0.34	−0.02	0.52**
CpG3	0.24	−0.23	0.25	0.30	0.39^*^	0.08	−0.23	−0.11	0.19	0.02	−0.07	0.03	0.30	−0.10	0.36	0.10	0.44*
CpG4	0.11	−0.04	0.18	0.17	0.27	0.05	0.07	0.13	0.11	−0.06	0.06	0.22	0.15	−0.17	0.39*	−0.18	0.50**
CpG5	−0.14	−0.18	0.13	0.16	0.16	0.08	−0.01	0.10	−0.06	−0.01	0.09	0.14	−0.08	0.21	0.02	−0.28	0.34
Mean CpG^+^	0.06	−0.12	0.24	0.17	0.30	−0.01	−0.13	−0.10	0.15	−0.10	−0.02	0.05	0.02	−0.04	0.28	−0.15	0.27

*Note.* Mean CPG^+^, mean of CpG1-5 sites; BIS, Behavioral Inhibition System; BAS, Behavioral Activation System. Bolded correlations remained significant after Bonferroni *p*-adjustment. *p < 0.05. **p < 0.01.

Relationship between discordances of MZ twins on categorial variables (alcohol use, cigarette use and drug abuse) and intrapair differences in DNAm.

The Mann-Whitney U-test showed significant differences in DNAm between concordant and discordant MZ twin pairs in alcohol consumption, with discordant twins had a significantly larger difference in the percentage of *MB-COMT* DNAm at CpG site 1 and CpG site 3 compared to concordant twin pairs (Table 5).

**TABLE 5:** Results of Mann-Whitney U-tests: Differences between concordant and discordant MZ twin pairs in risk behaviors.

DNAm difference (%)	Alcohol use			Cigarette use			Drug abuse		
	C *n* = 32		DC *n* = 14	C *n* = 41		DC *n* = 5	C *n* = 42		DC *n* = 4
	Median		*P*	Median		*p*	Median		*p*
CpG1	3.06	7.61	**0.02**	4.16	0.75	**0.03**	4.11	2.19	0.16
CpG2	1.93	1.52	0.86	1.77	0.42	0.21	1.75	1.70	0.84
CpG3	2.01	4.51	**0.03**	2.99	1.34	0.11	2.66	1.76	0.33
CpG4	1.99	2.48	0.50	2.07	1.82	0.35	2.29	0.22	**0.03**
CpG5	1.32	2.38	0.15	2.00	1.00	0.17	1.90	1.50	0.46
Mean CpG	1.66	3.12	0.06	2.49	0.68	**0.03**	2.27	0.61	0.09

Note. C, concordant; DC, disconcordant. *n* = number of MZ twin pairs. Bolded *p*-values remained significant after Bonferroni *p*-adjustment.

Somewhat surprisingly, MZ twin pairs reporting cigarette smoking for both twins had a difference in the percentage of *MB-COMT* DNAm that was significantly higher at CpG site 1 and in the mean of all CpG sites. Similarly, we found that *MB-COMT* DNAm difference between MZ twin pairs in which both twins reported drug use was significantly higher than for those in which only one twin did at CpG site 4. Thus, these results pointed to the greater intrapair DNAm differences in phenotypically concordant, than in discordant MZ twin pairs regarding cigarettes and drugs consumption.

## Discussion

In this study, a comprehensive range of indicators of impulsivity and lack of control is associated with DNAm percentages at five different CpG sites of the *MB-COMT* gene in MZ twins. The most important result is that the differences between MZ twins on DNAm at three of the five analyzed CpG sites in the promoter region of *MB-COMT* are associated with phenotypic indicators of lack of control. Although these associations are of moderate intensity, they deserve a more thorough consideration, which would facilitate the accumulation of knowledge necessary to understand the mechanisms underlying them.

The rRST, which includes stable responses to environmental stimuli, as sources of potential reward, punishment, or danger (Gray and McNaughton, 2000), is particularly important for the examination of epigenetic mechanisms. We found that phenotypic differences between MZ twins at Fight and Freeze are associated with their differences at methylation level. Twin studies consistently show that approximately half of the variance in all personality traits is genetically influenced (Takahashi et al., 2007; Kandler et al., 2010). It is plausible that the similarity of MZ in personality traits stems from the same genotype and shared environment, while all differences can be attributed to unshared environmental influences. Result that differences at Fight are associated with differences in *MB-COMT* DNAm levels at CpG site 4, may have important implications for etiology of aggression. It is quite possible that reactive aggression, defined by Fight, as a striking phenotypic indicator of lack of control, is influenced by environmental factors (Sypher et al., 2019), which determine epigenetic modulation on at least one DNAm marker. The association of the phenotypic differences between MZ twins at Freeze and differences in *MB-COMT* DNAm observed at CpG site 2, CpG site 3, and CpG site 4 is a particularly intriguing finding. Although Freeze, seemingly, cannot be easily associated with impulsive behavior, the importance of cognitive blockage in dealing with threatening stimuli should not be overlooked, since it can imply the perception of a lack of control over reactions and events (Smederevac et al., 2014). It is also possible that behavioral lack of control (Fight) includes some form of cognitive blocking (Freeze), which prevents the processing of broader information, narrowing the choice of possible reactions. Both Fight and Freeze systems underlie responses to the present threat, and although behaviorally different, both may be initiated by a similar experience. The perception of losing control can thus be manifested in a sudden and aggressive reaction to a threatening situation, but also in a more subtle way in the complete absence of reaction, i.e., freezing. Although Freeze and Fight are negatively correlated (Smederevac et al., 2014), they can have joint positive effects on certain behaviors (e.g., Sadiković et al., 2020). Since in our study both dimensions correlate positively with *MB-COMT* DNAm CpG site 4, the possibility of joint covariance of epigenetic changes in the domain of cognitive and behavioral reactions arises.

Besides self-report measures, differences between MZ twins in aggression was identified in specific aggressive behavior measured by experimental CRTT procedure as unprovoked direct aggression. Previous research using CRTT showed that shared and non-shared environmental factors, without genetic influences, almost equally contributed to the explanation of the initial level of aggression (Dinić et al., 2020) which could be linked to aggression in the 1st block of the CRTT scale. Thus, unprovoked aggression is partly influenced by the learned patterns developed in a shared, family environment. Significant associations were observed for *MB-COMT* DNAm MZ twin differences in CpG site 1 and CpG site 3 for block 1, which is in contrast to the association found with rRST Fight and CpG site 1. This implies a link to different aspects of aggression and different DNAm markers, CpG site 1 and 3 for direct unprovoked (CRTT) versus reactive (rRST Fight) aggression associated with CpG 4. If we consider that phenotypes result from specific combinations of genome composition, epigenetic components, and environmental influences (Cavalli & Heard, 2019), this result suggests that the effect of various environmental triggers could manifest in changes to DNAm at different sites associated with aggressive responses.

At WCST, as a complex measure of executive functions, MZ twins differ in Failures to maintain set and Non-perseverative errors, which are associated with different DNAm levels at CpG site 1. Failures to maintain set, as a measure of distractibility (Barceló and Knight, 2002) or cognitive flexibility (Greve et al., 2005), refers to the number of failures in sorting cards according to the sorting rule, due to a change of direction before the rules require it. Non-perseverative errors refer to a qualitative change in the search for the right strategy, which indicates the flexibility, yet ineffective, of cognitive style (Greve et al., 1996). As indicators of executive dysfunction, these measures may be associated with impulsivity and lack of control (Bickel et al., 2012). The correlation with CpG site one, which is also associated with the aggressive reactions, provides additional indirect evidence of the relationship between executive function and aggression. Therefore, Failures to maintain set may represent an aggressive response to a structured task with positive and negative feedback, an integral part of the WCST administration, which opens a new path for examining the etiology of executive dysfunctions. Namely, previous findings have shown large effect sizes related to association between failures to maintain set at WCST and aggression/violence (Burgess, 2020).

Despite the small sample size, this study identified discordant MZ twin pairs in relation to substance abuse as a form of risk behavior. Alcohol abuse is significantly associated with the level of DNAm at CpG site 1 and CpG site 3, confirming the importance of this aspect of methylation changes for dysfunctional aspects of impulsivity, which are reflected in the inability to resist temptation. Namely, this site showed the largest number of associations with different phenotypic traits, which include cognitive and behavioral indicators of lack of control. Although cigarette consumption is significantly associated with the level of DNAm at CpG site 1 and drug consumption with the level of DNAm at CpG site 4, the direction of the differences indicates an artifact due to the extremely small number of discordant twins on these phenotypic features. Also, it is possible that the epigenetic mechanisms underlying the use of alcohol and cigarettes, as more common forms of behavior, are significantly different from those of drug abuse, but these should be further investigated in future studies.

Interestingly, CpG site 2 and CpG site 5 are not relevant for any of the examined phenotypic traits. It is possible that each of the sites has a specific role in epigenetic mechanisms, which determines unique patterns of behavior. The most consistent links with different lack of control indicators exist for CpG 1 and CpG 3, while CpG 4 is related only to Fight. One of the most important implications of the results of this study is the indication that there are common epigenetic patterns for personality traits, cognitive functions, and behaviors. The highest level of methylation was registered at CpG1, associated with phenotypic differences among MZ pairs on five indicators of lack of control. Two of them belongs to measures of executive functions, one to aggressive reactions and two are behavioral measures of risky behaviors-alcohol and cigarettes abuse. In general, hypermethylation is associated with decreased gene expression, which is also related to *MB-COMT* (Abdolmaleky et al., 2006). Therefore, a potential risk mechanism for lack of control may lead to reduced methylation and higher levels of *MB-COMT* expression. In other words, increased COMT activity can lead to lower levels of dopamine in synapses. In this context, the role of CpG1 may be particularly important. In our study, it is bound to the P53 binding site CCCGGG (Yang & Festing, 2001) which protects the organism from the spread of an aberrant genetic signal (Jacobs et a., 2006) and is associated with neurological development and mental illnesses such as schizophrenia (Warburton et al., 2015). Also, CpG2 is bound to the SP1 binding dynamics (GGGCGG), which affects the expression of many genes including genes linked to mental diseases or functions (Abdolmaleky et al., 2006; Hasegawa, and Struhl, (2021).

It is plausible to assume that epigenetic modulations on the *MB-COMT* gene associated with phenotypic differences on impulsivity indicators in MZ twins are probably due to environmental influences. Given the similar environment experienced by MZ twins during development, differences in epigenetic events may be explained by unshared environmental factors (Wong et al., 2005). Previous research suggests that gene expression pattern can be strongly affected by environmental factors (Sutherland and Costa, 2003; Roth, 2013), so all unshared experiences can contribute to the discordance of MZ twins, through mechanisms such as DNAm. Although they cannot be viewed as a direct replication of previous studies, these results partially support the findings that indicate the importance of DNAm on the *MB-COMT* gene for individual differences in impulsivity and lack of control (Nielsen et al., 2012; van der Knaap et al., 2014; Lewis et al., 2019).

One important limitation of this study is the small sample size, which limits the relevance of our findings. However, this limitation is partially compensated by many impulsivity indicators and epigenetic analyzes, generated on one sample of MZ twins. We also observed some smaller effects that were no longer statistically significant after the correction for multiple comparisons; however, these can provide a base for future studies. Previous research has shown that small effects could have substantial consequences on the identification and understanding of the actual determinants of complex psychological phenomena (Götz et al., 2022). Also, one of the major limitations of this study is lack of concurrent gene expression analysis to portray functional impacts of DNA methylation alterations, which should certainly be included in future replication studies. The epigenetic effects of the *MB-COMT* gene on personality, risk behaviors, and cognitive processes suggest its pleiotropic role in the regulation of various phenotypic features. Namely, associations between phenotypic differences and differences in methylation level represent a contribution to the accumulation of evidence on the influence of the environment on the structure of the epigenome. The current results highlight the importance of studying gene methylation in the context of their genetic environment and the impact on phenotypic traits associated with lack of control. However, it is still difficult to determine how these changes affect gene expression and thus modulate phenotypes, since functional analyzes of these CpG sites are not yet associated with a wider spectrum of phenotypic characteristics.

## Data Availability

The data that support the findings of this study are openly available in the OSF repository: https://osf.io/694yn/.
